# Regulatory mechanisms and biosynthesis of chlorogenic acid in *Lonicera japonica*: insights from tissue culture and inducer treatments

**DOI:** 10.3389/fpls.2025.1567140

**Published:** 2025-04-24

**Authors:** Jiali Cheng, Yuan Chen, Fengxia Guo, Pengbin Dong, Chunyan Zhou, Wei Liang, Hongyan Wang

**Affiliations:** ^1^ College of Agronomy, College of Life Science and Technology, State Key Laboratory of Aridland Crop Science, Gansu Agricultural University, Lanzhou, China; ^2^ College of Economics and Management, Hexi University, Zhangye, China

**Keywords:** *L. japonica*, tissue culture, chlorogenic acid, elicitors, synthetic biology

## Abstract

Plant tissue culture is a fundamental and widely applied technique in plant biology and agriculture. In medicinal plant research, tissue culture plays an indispensable role in the conservation of endangered species, the rapid propagation of valuable resources, the preservation of germplasm, and the production of secondary metabolites. As a representative medicinal plant of the *Lonicera* genus, *L. japonica* is widely utilized worldwide due to its significant economic, ecological, medicinal, and ornamental value. By using tissue culture technology, it is possible to significantly enhance the production of secondary metabolites in *L. japonica* and effectively alleviate resource shortages, providing a new approach for its sustainable utilization. This review summarizes the recent research progress on *L. japonica* in the field of tissue culture, covering aspects such as direct organogenesis, indirect organogenesis through callus tissues, protoplast culture, hairy root culture, and polyploid culture. Additionally, the biosynthetic pathway of chlorogenic acid was explored in detail, and the mechanism of action of inducers in plant cells was analyzed. The study focused on the potential regulatory mechanisms of inducers on chlorogenic acid. Eventually, the future development trends of medicinal plant biotechnology are envisioned, aiming to provide a broader perspective for the in-depth study of medicinal plants and to promote continuous development and innovation in this field.

## 1 Introduction

**Figure 1:**
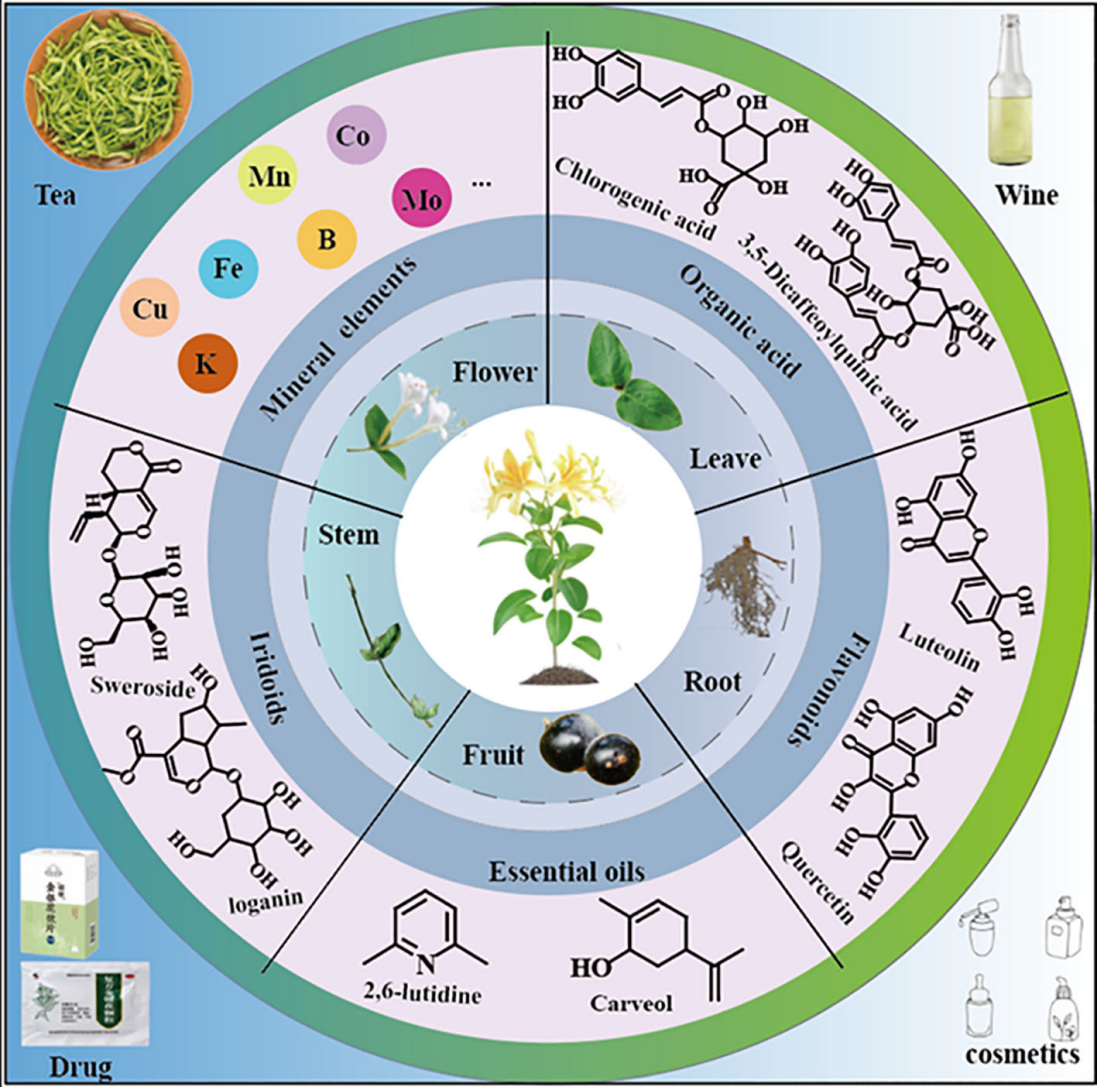
The organs, main components and processing products of *L. japonica*.

*Lonicera* are usually erect or dwarf shrubs, with a few species in the form of small trees, occasionally as twining vines, and are deciduous or evergreen (Liu et al., 2022). Its corolla is richly colored, commonly including white yellow, pale pink or purplish-red. The fruits are berries, with colors ranging from red to bluish-black or black. The *Lonicera* genus comprises approximately 200 species, primarily distributed across temperate and subtropical regions of North America, Europe, Asia, and northern Africa. In Asia, its distribution extends as far south as the Philippines and southern Malaysia (Jacobs et al., 2009; Park et al., 2019). In China, approximately 98 species, including 4 subspecies, 18 varieties, and 1 forma, are widely distributed across various provinces, with the highest abundance in the southwestern regions (Hsu and Wang, 1988; Yang et al., 2019a). *Lonicera* has significant medicinal value, with *L. japonica* being the most renowned. It is listed among the 35 valuable Chinese medicinal herbs by the Administration of Traditional Chinese Medicine of China. Surveys indicate that approximately 18 types of flower buds from this genus are used as Chinese herbal medicine commodities (Hsu and Wang, 1988) (**Table 1**). *L. japonica* contains diverse chemical constituents, including flavonoids, phenolic acids, cyclic enol ether terpene glycosides, volatile oils, and other bioactive substances, of which chlorogenic acid (CGA) and lignans are the main active components (Shang et al., 2011; Yang et al., 2019b). These components have various pharmacological effects such as antioxidant, anti-inflammatory, antibacterial and antiviral (Su et al., 2021; Liu et al., 2016). Therefore, *L. japonica* has been widely used in the fields of traditional Chinese medicine, functional foods, nutraceuticals and cosmetics (Fang et al., 2022; Liu et al., 2023) (**Figure 1**). Additionally, *L. japonica* has gained significant attention as a natural plant resource owing to its abundant bioactive compounds and high safety.

**Table 1:** *Lonicera* species of medicinal plants.

Genus	Subgenus	Group	Species	Locality	Growing environment	References
*Lonicera*	*Subgen. Chamaecerasus* (Linn.) Rehd.	*Sect*. *lsika*	1. *L. lanceolata* Wall	Eastern and western regions of Sichuan, the northeastern to northwestern and southwestern regions of Yunnan, the eastern to southern regions of Tibet, Nepal to Bhutan.	Grows in coniferous and broad-leaved mixed forests, fir forests, or shrublands at forest edges, at an altitude of 2,000-3,900 meters.	Zhao et al. (2018); Hsu and Wang (1988)
			2. *L. hispida* Pall	western Hebei; Shanxi; southern Shaanxi; southern Ningxia (Jingyuan, Longde); central to southern Gansu; eastern Qinghai; Xinjiang; western Sichuan; northwestern Yunnan; and eastern to southern Tibet; Mongolia, Central Asia (former Soviet regions), and northern India	Grows in mountain forests, shrublands at forest edges, or alpine meadows at elevations of 1,700–4,200 meters, reaching up to 4,800 meters in Sichuan and Tibet.	Hsu and Wang (1988)
		*Sect*. *Coeloxylosteum*	3*. L. maackii* (Rupr.) Maxim	Changping, Beijing; eastern Heilongjiang, Jilin, and Liaoning; southern Hebei; southern Shanxi; Shaanxi; southeastern Gansu; eastern and southwestern Shandong; Jiangsu; Anhui; northern Zhejiang; Henan; Hubei; northwestern and southwestern Hunan (Xinning); northeastern Sichuan; Guizhou (Xingyi); eastern to northwestern Yunnan; and Tibet (Gyirong); Korea; Japan; and the Russian Far East.	Grows in forests or shrublands near streams at forest edges, at elevations up to 1,800 meters (reaching 3,000 meters in Yunnan and Tibet)	Sun et al. (2023); Hsu and Wang (1988)
		*Sect. Nintooa*	4*. L. crassifolia* Batal	Southwestern Hubei; northwestern Hunan (Sangzhi); southeastern and southwestern Sichuan; western Guizhou (Bijie) and northern Guizhou (Daozhen); as well as Yunnan (Malipo).	Found beside streams or in moist forest edge rock walls or crevices, at altitudes of 900-1700 (-2300) meters.	Hsu and Wang (1988)
			5. *L. acuminata* Wall	Southern part of Shaanxi; southeastern Gansu; southern Anhui; Zhejiang (Longquan, Qingyuan, Suichang); western and northeastern Jiangxi; Fujian (Chongan); Taiwan; western Hubei; northwestern Hunan; northern Guangdong (Ruyuan); northeastern to northern Guangxi; Sichuan; Guizhou; northeastern to northwestern and western Yunnan; southeastern to southern Tibet; eastern Himalayas through Myanmar to Sumatra, Java, Bali, and the Philippines.	Grows in forests, open areas between forests, or in shrublands on hillsides and in valleys, at altitudes of (500-) 1000-3200 meters.	Sun et al. (2023); Yang et al. (2022); Hsu and Wang (1988)
			6*. L. inodora* W.W. Smith	Western Yunnan (Tengchong) and southeastern Tibet (Motuo).	Found in shrubbery on rocky hills or in montane broadleaf forests, at an elevation of 1700-2900 meters.	Hsu and Wang (1988)
			7. *L. pampaninii* Levl	Southern part of Anhui (Huangshan, Qingyang); Zhejiang, the northwest and northeast of Jiangxi; northern Fujian; southwestern Hubei; Hunan; northern Guangdong; northeastern and southeastern Guangxi (Luchuan); southeastern Sichuan; eastern to northern Guizhou; and southern Yunnan (Jianshui).	Found in the understory or shrubbery, at an elevation of 150-750 (-1400) meters.	Jiang et al. (2021); Hsu and Wang (1988)
			8*. L. dasystyla* Rehd	Guangdong (Dinghu Mountain, Zhaoqing) and northern Guangxi; northern Vietnam.	Grows in shrubbery near water, at altitudes below 300 meters.	Hsu and Wang (1988)
			9. *L. japonica* Thunb	Distributed in all provinces of China except for Heilongjiang, Inner Mongolia, Ningxia, Qinghai, Xinjiang, Hainan, and Tibet; also found in Japan and North Korea.	Found in shrubbery or sparse forests on hillsides, rock piles, at the foot of mountains, or along village fences, at altitudes up to 1500 meters.	Hsu and Wang (1988); Zhang et al. (2022b)
			10*. L. hypoglauca* Miq	southern part of Anhui; Zhejiang; Jiangxi; Fujian; northern and central Taiwan; southwestern Hubei; western to southern Hunan; Guangdong (except the southern region); Guangxi; eastern and southeastern Sichuan; northern, southeastern; and southwestern Guizhou; northwestern to southern Yunnan; Japan.	Grows in shrubbery or sparse forests at altitudes of 200-700 meters (up to 1500 meters in the southwest)	(Li et al., 2024b); Hsu and Wang (1988)
			11. *L. macrantha* (D.Don) Spreng	Southern Zhejiang; western Jiangxi; Fujian (Nanping); southwestern Hunan; Guangxi; northeastern Sichuan (Nanjiang); southeastern Sichuan (Xingwen, Jiangbei, Xiushan); Guizhou; and southeastern and western Yunnan.	Grows in hilly or valley forests or shrublands, at altitudes of 350-1250 meters, reaching up to 1800 meters in Yunnan.	Hsu and Wang (1988)
			12. *L. longgiflora* (Lindl.) DC	Southern Guangdong; Hainan; and Yunnan (Maguan).	Found in open forests or sunny areas along mountain roads, at altitudes reaching up to 1700 meters.	Hsu and Wang (1988)
			13*. L. macranthoides* Hand.-Mazz	Southern part of Anhui; Zhejiang; the western part of Jiangxi; the northwestern part of Fujian; the southwestern part of Hubei; the southern to western parts of Hunan; Wengyuan in Guangdong; the northeastern part of Guangxi; the southeastern part of Sichuan; and the eastern and northwestern parts of Guizhou.	Grows in mixed forests or shrubs beside streams in valleys, on hillsides, or mountain tops at altitudes ranging from 500 to 1800 meters.	Hsu and Wang (1988); Chen et al. (2023)
			14. *L. buchananii* Lace	Western part of Yunnan (Yingjiang) and northern part of Myanmar.	Grows in an altitude of approximately 200 meters.	Hsu and Wang (1988)
			15. *L. rhytidophylla* Hand.-Mazz	Southwest of Jiangxi; the central and northern parts of Fujian; the central and western parts of Fujian; the southern part of Hunan; Guangdong; and the northeastern part of Guangxi.	Grows in mountain shrubs or forests, at an altitude of 400-1100 meters.	Hsu and Wang (1988)
			16. *L. confusa* (Sweet.) DC	Ho Chi Minh, Vietnam; Danzhou, Hainan, China; Haikou, Hainan, China.	Grows on hillsides, in mixed forests and shrubs, as well as along open plains, roadsides, or riverbanks, at altitudes up to 800 meters.	Sun et al. (2023); Hsu and Wang (1988)
			17. *L. similis* Hemsl	Southern Shaanxi; southern Gansu; northwestern and southwestern Zhejiang; Fujian; western Hubei; western Hunan; Du’an in Guangxi; northern, eastern; and southwestern Sichuan; western to northern Guizhou; and eastern to northern Yunnan; Myanmar.	Grows along mountain valley streams or in shrubs and forests on sunny slopes at elevations of 550–1,600 meters, reaching up to 2,200 meters in Sichuan and Yunnan.	Wei et al. (2021); Hsu and Wang (1988)
	*Subgen. Lonicera*		18*. L. tragophylla* Hemsl	Southwestern Hebei; southern Shanxi; central to southern Shaanxi; southern Ningxia and Gansu; western and southern Anhui; northwestern and southern Zhejiang (Longquan); northwestern Henan; western and eastern Hubei (Luotian); Sichuan and northern Guizhou.	Grows in understory forests, shrublands, or rock crevices near riverbanks at elevations of (700–)1,000–2,000(–3,000) meters.	Sun et al. (2023); Hsu and Wang (1988)

The main sources of medicinal plants include wild and cultivated resources. However, overexploitation and land degradation have led to a sharp decline in the availability of wild resources. Field cultivation is time-consuming and labor-intensive, with continuous cropping often resulting in failures due to increased disease incidence (Shi et al., 2018; Sun et al., 2011). These problems exacerbate the scarcity of medicinal plant resources and raise the risk of extinction of endangered species. According to the National Economic Forestry Association (NEFA), the global market demand for *L. japonica* has exceeded 20 million kilograms, with a value of $860 million, making it one of the most economically valuable medicinal plants (Lai et al., 2022). However, the long cultivation period of *L. japonica*, its susceptibility to pathogens and replanting diseases, and the instability of active compound content in cultivated plants severely limited farmers in meeting the growing market demand (Jia and Yan, 2024; Pan et al., 2021a).

To address these challenges, plant tissue culture technology has developed rapidly and is widely regarded as a critical tool for plant regeneration, rapid propagation of pathogen-free and disease-free materials, and efficient production of secondary metabolites. This technology offers significant advantages, including enhanced plant responses to biotic and abiotic stresses, as well as substantial improvements in the levels of desired traits and specific compounds (Tomasiak et al., 2022). Research on plant tissue culture and cell culture began in the late 19th century, when Haberlandt first introduced the concept of cell culture and pioneered the use of artificial media for the cultivation of isolated plant cells (Haberlandt, 1902). In 1934, White achieved a major breakthrough in the cultivation of isolated roots by establishing the first actively growing asexual line from isolated tomato roots, and discovered the important role of the B vitamins B1, B6 and niacin in plant growth (White, 1934). In 1965, Vasil and Hildebrandt successfully regenerated whole plants from a single isolated cell culture, scientifically validating the theory of plant cell totipotency (Vasil and Hildebrandt, 1965). During the 1960s, microspore and protoplast cultures were successfully provided. Subsequently, a large number of studies have demonstrated that undifferentiated plant cells (e.g. callus and cell suspensions) have broad application prospects in the large-scale production of secondary metabolites for pharmaceuticals and cosmetics (Xu et al., 2023; Asyakina et al., 2021).

**Figure 2:**
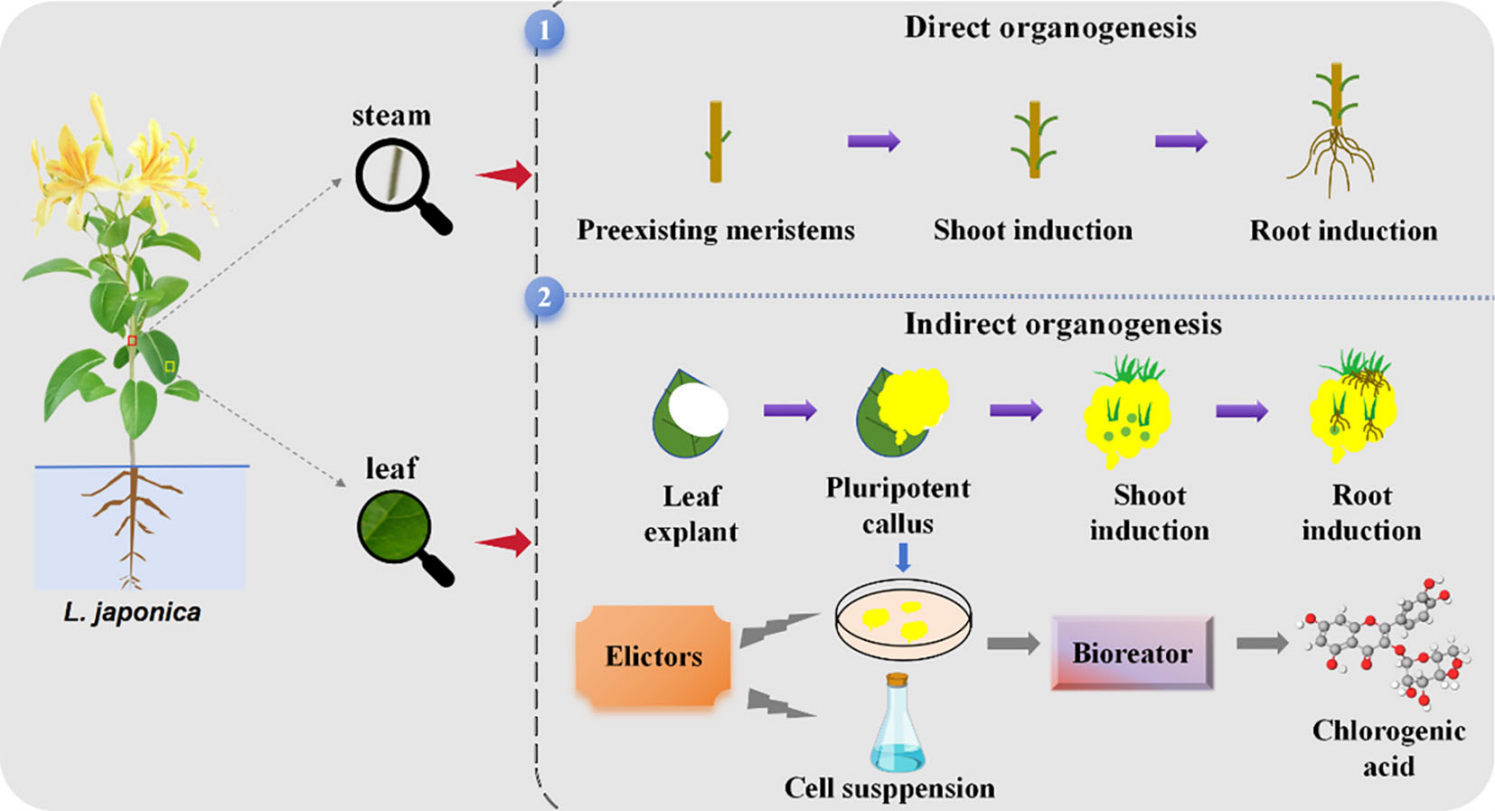
Direct and indirect organogenesis pathway processes in *L. japonica*.

Recent studies have shown that phenolic acids can be successfully obtained from *L. japonica* employing *in vitro* tissue cultures. This paper reviews the research progress on tissue culture of *L. japonica* conducted both domestically and internationally, covering the direct and indirect organogenesis pathways (as shown in **Figure 2**), protoplast culture, cell suspension culture and hairy root culture techniques of *L. japonica*. Additionally, the biosynthetic pathway of CGA, microbial transformation and metabolic regulation engineering were discussed in detail. Finally, it explores future trends in medicinal plant biotechnology, offering new perspectives and potential for further research and broader applications in this field.

## 2 *In vitro* culture techniques of *L. japonica*

**Figure 3:**
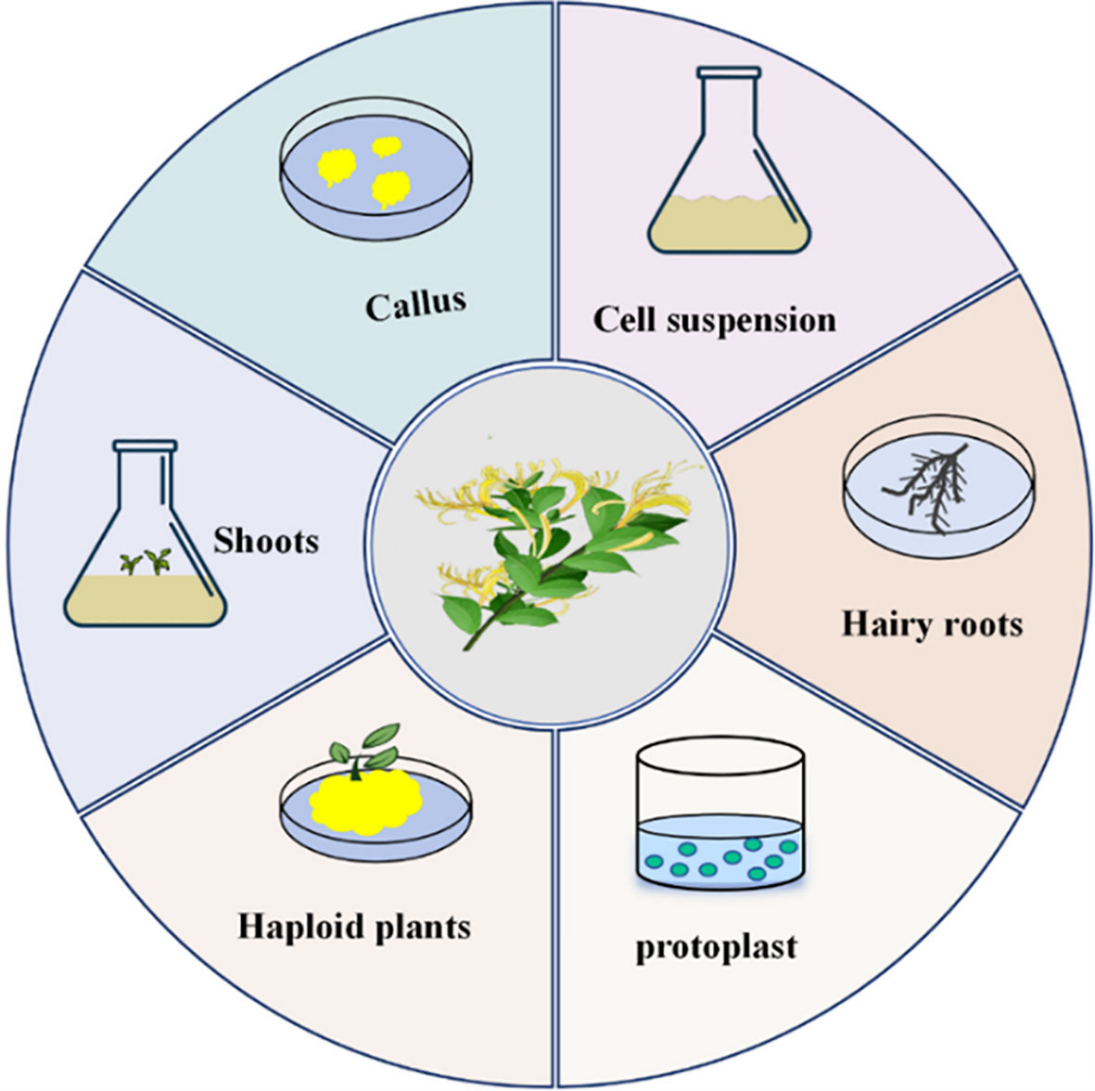
Types of *L. japonica* tissue culture.

Tissue culture can be classified based on the purpose of cultivation and the source of explants. In the study of *L. japonica*, several key processes of tissue culture based on cells and organs have been established (**Figure 3**), providing technical support for the development of *L. japonica* resources and further promoting the sustainable utilization of this species.

### 2.1 Direct organogenesis of *L. japonica*

The direct organogenesis pathway induces the existing organ primordia of explants to directly form adventitious shoots or roots, without the presence of callus morphology throughout the induction process (Kong et al., 2021).

In the direct organogenesis pathway, shoot induction is typically based on MS medium, with 6-BA (a cytokinin) as the primary exogenous hormone. Research by Xun (2004). indicated that the optimal medium for inducing multiple shoots and subculturing of *L. japonica* is MS + 6-BA 0.5 mg/L + IBA 0.05 mg/L, the optimal rooting medium is MS + IBA 1.0 mg/L + NAA 0.05 mg/L. The study also found that browning of explants is associated with the activity of polyphenol oxidase (PPO) in the tissue. PPO catalyzes the oxidation of phenolic compounds, resulting in the formation of dark-colored substances that inhibit the activity of other enzymes and cause toxicity to the explants. To mitigate the toxicity caused by browning, it is recommended to perform continuous subculturing during the early stages of cultivation. Gao and Dong (2018) used one-year-old stem segments of wild *L. japonica* as explants to determine the optimal medium for adventitious bud induction as MS + 6-BA 1.5 mg/L + NAA 0.12 mg/L, the best subculture medium for propagation as MS + 6-BA 1.5 mg/L + NAA 0.02 mg/L, and the optimal rooting induction medium as 1/2 MS + IBA 2.5 mg/L + NAA 0.2 - 0.6 mg/L. Yang et al. (2017) used young stems as explants and determined the optimal medium for adventitious bud induction as MS + 6-BA 2.0 mg/L + IBA 0.01-0.05 mg/L, the most suitable medium for subculturing plantlets as MS + 6-BA 1.0 mg/L + IBA 0.01-0.05 mg/L, and the best rooting induction medium as 1/2 MS + IBA 2.0 mg/L + NAA 0.5 mg/L. Wang et al. (2013) showed that the apical bud shoot induction medium of *L. japonica* was MS + KT 1.0 mg/L + 6-BA 2.0 mg/L + NAA 0.1 mg/L, the proliferation medium was MS + KT 1.5 mg/L + 6-BA 1.5 mg/L + NAA 0.3 mg/L, and the rooting medium was 1/2 MS + NAA 2.0 mg/L + IAA 0.15-0.20 mg/L. However, Yang et al. (2003) used B5 medium in the tissue culture of Mongolian *L. japonica* and found that B5 + 6-BA 2.0 mg/L + KT 0.5 mg/L + IAA 1.0 mg/L + LH 1000 mg/L had a significant effect on the induction of axillary buds.

### 2.2 Callus tissue

To date, various explants, including stems, leaves, buds, flower buds, and roots, have been widely used for inducing callus tissue in *L. japonica*, with leaves and stem segments being the most commonly used explants. Typical callus induction cultures generally use Murashige and Skoog (MS), B5, or LS basal media containing 3% sucrose and various concentrations of plant growth regulators (PGRs). Researchers have explored the effects of plant growth regulators, such as cytokinins (6-BA, KT, ZT), among which 6-BA is the most commonly used, with an optimal concentration typically ranging from 1.0 to 2.5 mg/L. Auxins play a critical role in callus induction by promoting the dedifferentiation of cells to form callus tissue and maintaining a continuous state of division and proliferation. NAA is commonly used in callus induction, typically at concentrations ranging from 0.01 to 1 mg/L. The culture temperature is typically 25°C, with a photoperiod of 14 h per day and a light intensity of approximately 2000 lux. Georges et al. (1993) found that the optimal medium for inducing *L. japonica* callus was LS + BA 2.7 μM + NAA 10.7 μM. However, substituting 6-BA with 2,4-D led to rapid browning of the explants, significantly reducing the induction efficiency. Liu and Qian (2007) optimized the callus induction medium by adjusting the concentrations of exogenous hormones in MS basal medium, resulting in the ideal formulation of MS + 6-BA 0.1 mg/L + NAA 1.0 mg/L. Their study showed that 6-BA was most effective at concentrations of 0.1 mg/L and 0.5 mg/L, with the former achieving an induction rate of 95.1%. NAA and IAA were both effective within the range of 0.5 to 1.5 mg/L, with NAA at 1.0 mg/L yielding the highest induction rate (97.5%), whereas IAA at the same concentration only reached 88.3%. Xiang and Gao (2007) found that although MS medium could induce callus tissue, its growth was poor due to the possible inhibitory effect of ammonium salts. In contrast, B5 medium, with a lower ammonium salt content, significantly promoted callus growth when supplemented with IAA 0.5 mg/L, BA 2.0 mg/L, and KT 0.5 mg/L. Song and Liang (1987) used young *L. japonica* stems and leaves as explants, and added 1 ppm 2,4-D and 0.1 ppm KT to the MS medium. The results showed that the callus grew well, with the content of CGA and isochlorogenic acid reaching approximately 50% of that in the flower buds. The callus tissues are summarised in **Table 2**.

**Table 2:** Callus culture of *L. japonica*.

Time	Explant	Medium	PGRs	Cultivation Conditions	Result	References
1987	Young leaf & stem	MS	1 ppm 2,4-D+0.1 ppm KT	The explants were sterilized with 0.1% HgCl_2_ and cultured at 25°C.	The callus tissue exhibited rapid growth and strong proliferative capacity. CGA and isochlorogenic acid were detected in the callus tissue, with a content of approximately 2.5%.	Song and Liang (1987)
1991	Leaf	MS	2.3 µM TDZ	Maintained in darkness for 3 weeks at 23°C, following which the explants were transferred to a medium without auxin and under low intensity illumination (5.8 pmol m^-2^ s^-1^).	Under auxin-free culture conditions, callus formation was observed when the TDZ (N-phenyl-N’-1,2,3-thiadiazol-5-ylurea) concentration reached 0.045 μM. As the TDZ concentration increased to 2.3 μM, callus growth became significantly more vigorous.	Cambecèdes et al. (1991)
1991	Leaf protoplasts of axenic shoot cultures	MS	2.0 mg/L NAA+0.2 mg/L 6-BA	Cultures were kept at 25°C, with a 16/8 h light/dark photoperiod of 2000 lux from cool white fluorescent tubes.	MS medium with 2.0 mg/L NAA and 6.2 mg/L BAP, with the production.	Ochatt (1991)
1993	Leaf, steam & root	LS	10.7 µM NAA+2.7 µM 6-BA	Cultures kept at 25°C under a 16 h light photoperiod of 54 µmol s ^-1^ m ^-2^ (cool white fluorescent tubes).	Leaves were the most responsive, with 100% of explants giving; Medium containing 10.7 µM NAA and 2.7 µM 6-BA (under photoperiodic illumination) gave the best results, while media with 2,4-D led to a rapid necrosis of explants.	Georges et al. (1993)
2003	Stem segment	B5	2.0 mg/L 6-B +0.2-0.5 mg/L KT+0.5-1.0 mg/L IAA+1000 mg/L LH	The temperature was maintained at 25 ± 2°C, with a relative humidity of 60%-75%. Artificial lighting was provided for 12-14 h/d with light intensity ranging from 1500 to 2000 lx.	The callus tissue exhibited relatively rapid growth.	Yang et al. (2003)
2004	Stem	MS	2.0 mg/L 6-BA +0.1 mg/L NAA	Two 40 W fluorescent lamps were used, with a light duration of 10-12 h, and the temperature was maintained between 24°C and 28°C.	MS medium was found to be the optimal basic medium, with callus formation occurring most rapidly, taking only 18 d.	Li and Sun (2004)
2005	Stem segment	MS	1.0 mg/L 6-BA +0.2 mg/L NAA	The light intensity was maintained at 1500-2000 lx, with a light duration of 12-14 h/d, and the temperature was controlled between 24°C and 28°C.	After 5-10 d, loose white callus tissue began to form at the base of the seedlings. Around 30 d, the white callus gradually turned green, became more compact, and developed a granular surface.	Qiu and Tan (2005)
2007	Bud, leaf & stem	MS	0.1 mg/L 6-BA +1.0 mg/L NAA	The samples were first cultured in the dark for 24 h, followed by incubation at 25°C under light intensity of 1000-1500 lx with a photoperiod of 12 h/d.	Using buds as explants yielded the best results for callus induction, as it not only produced a large quantity of callus but also resulted in faster induction.	Liu and Qian (2007)
2007	Bud, steam & leaf	B5	2.0 mg/L 6-BA +0.5 mg/L IAA +0.5 mg/L KT	The light duration was 16 h, with a light intensity of 1000 lx, and the culture temperature was maintained at (25 ± 1) °C.	B5 medium was used as the basic medium, and both buds and leaves induced vigorous callus growth.	Xiang and Gao (2007)
2010	Bud & leaf	MS	0.01 mg/L 6-BA+2 mg/L NAA+0.5 mg/L 2,4-D	The temperature was maintained at 25 ± 2°C, with a light duration of 14 h/d and a light intensity of 2000 lx.	The best explants were buds and the best induction medium was MS+NAA 2 mg/L+6-BA 0.01 mg/L+2,4-D 0.5 mg/L, the inducing ratio could reach 100%.	Liu (2010)
2011	Stem segment	MS	0.8 mg/L 6-BA +0.1 mg/L NAA	The culture temperature was maintained at (25 ± 1) °C, with a light duration of 16 h/d and a light intensity of 30-40 μmol m^-^²·s^-1^.	Adventitious buds emerged from the callus tissue at the base, with an average proliferation rate exceeding 3.	Xu et al. (2011)
2012	Bud	1/2MS	1.0 mg/L 6-BA +0.1 mg/L NAA	The light intensity was 2000 lx, the temperature in the chamber was adjusted to (25 ± 2) °C, and the humidity ranged from 70% to 80%.	Adventitious buds emerged from the callus tissue at the base.	Li et al. (2012)
2013	The bottom of the flower bud	MS	0.01 mg/L 6-BA +2.0 mg/L NAA +0.5 mg/L 2,4-D	The temperature was (25 ± 2) °C, with a light intensity of 2000 lx and a photoperiod of 14 h/d.	The callus tissue grew rapidly, with a large quantity, loose and moist texture, and bright color.	Shan et al. (2013)
2015	Leaf	MS	1.5 mg/L 6-BA+1.0 mg/L NAA	The samples were first cultured in the dark for 24 h, then transferred to a 25°C incubator for further cultivation.	The callus tissue exhibited vigorous growth.	Ren et al. (2015)
2016	Bud	MS	0.15 mg/L 6-BA +1.5 mg/L NAA+1.0 mg/L 2,4-D+0.75 mg/L KT	The culture temperature was 25 ± 2°C with 60% humidity. In the first stage, the samples were cultured in the dark for 9 d. In the second stage, a light/dark alternation cycle was applied with a light intensity of 2000 lx and a photoperiod of 14 h/d.	The callus tissue was pale yellow, with a loose texture and vigorous growth. The growth curve followed an S-shape, with a growth rate of (21.62 ± 0.54) g per bottle after 24 d. Each 100 g of callus tissue contained 57 mg of CGA.	Hu et al. (2016)
2018	Stem segment	MS	1.5 mg/L 6-BA+0.2 mg/L NAA+1.0 mg/L KT	Under light culture conditions, the light intensity was 1200 lx. For dark culture, the inoculated materials were placed on a culture rack and covered with black boxes to prevent light exposure.	The callus tissue exhibited the highest induction rate and vigorous growth.	Heng et al. (2018)
2022	Young leaf	MS	4.0 mg/L 2,4-D	The culture temperature was 25°C, with a light intensity of 2500 lx and a photoperiod of 12 h/d.	At this concentration, the callus induction rate was the highest, and the callus tissue appeared greener, making it the optimal concentration for inducing callus in *L. japonica*.	He et al. (2022)

### 2.3 Research progress on the culture of *L. japonica* suspension cells

In recent years, with the rapid development of biotechnology, cell suspension culture has been widely applied in physiology, biochemistry, cytology, developmental biology, genetics and molecular biology due to its advantages such as rapid cell proliferation, large-scale culture capability, and uniformity of cultured materials (Moscatiello et al., 2013; Owis et al., 2016). Different from animal cells, plant cell suspension culture can establish stable culture systems without malignant mutation, As a result, they have been widely used in studies of metabolism (Popova et al., 2023; García-Pérez et al., 2021; Meyer and Fricker, 2002), cell cycle (Fabian-Marwedel et al., 2002; Kobayashi et al., 2021), and in the research of cellular biology (Yin et al., 2024; Moniruzzaman et al., 2021; Menges et al., 2003) and other cellular processes (Errico et al., 2025; Zagorskaya and Deineko, 2017; Hosseini and Mulligan, 2002). Kim et al. (2003) conducted embryogenic cell suspension culture of *L. japonica* using MS medium supplemented with 2,4-D at a concentration of 4.52 μmol/L, achieving an induction rate of 46.7%. After inoculation of embryonic cell suspension into MS solid medium, a large number of somatic embryos were obtained, with a regeneration rate of 68.0%. Liu (2010) used yellowish-white, small-grained, loosely structured and fast-growing callus as explants and transferred them into liquid medium with MS + 6-BA 1.5 mg/L + KT 0.75 mg/L + NAA 0.5 mg/L, established a cell suspension culture system, and investigated the physiological and biochemical changes of cells during the cultivation process. Hu et al. (2019) transferred induced callus into liquid MS medium containing BA (1.5 mg/L), NAA (0.2 mg/L), and 2,4-D (0.1 mg/L). The study revealed that the cell growth curve followed an “S” pattern, with the biomass reaching its peak on day 12. Additionally, the suspension culture was found to contain major secondary metabolites, including 3,5-di-O-caffeoylquinic acid, 3-O-caffeoylquinic acid, 4,5-di-O-caffeoylquinic acid, and 3,4-di-O-caffeoylquinic acid. Hu (2016) successfully established a *L. japonica* cell suspension culture system by inoculating 0.2 g/mL callus into liquid MS medium supplemented with 6-BA (1.4 mg/L), NAA (0.5 mg/L), and 2,4-D (0.7 mg/L) under dark conditions for 7-10 days. The cell biomass peaked on the 10th day, with 826.7 mg of CGA per 100 g of dry cells. Further studies demonstrated that CGA and isochlorogenic acid A exhibit significant nitrosation inhibitory activities, which are influenced by changes in the reaction environment. Wang et al. (2022) reported that after 18 days of suspension culture, the fresh cell weight reached 676.0 ± 12.66 g/L, which was 13.52 times the initial inoculation amount. Horiike and Ohshiro (1997) isolated six curdione derivatives from the cell suspension culture of *L. japonica*, including (2S)-2-hydroxycurdione, (2R)-2-hydroxycurdione, (8S)-6-hydroxycurdione, (2R, 8S)-8-hydro-2-hydroxycurdione, (1S, 10S)-1,10-epoxycurdione, and (1R, 10R)-1,10-epoxycurdione. Yamamoto et al. (1999) discovered that cell suspension cultures of *L. japonica* do not produce iridoid or secoiridoid glycosides. However, the cells can utilize 7-deoxyloganin 7-hydroxylase to convert 7-deoxyloganin into loganin and secoiridoid glycosides. Suspension cultures are summarized in **Table 3**.

**Table 3:** Cell suspension culture of *L. japonica*.

Time	Explant	Medium	PGRs	Cultivation Conditions	Result	References
1997	Young leaf	B5	0.1 mg/L 2,4-D+0.2 mg/L BA	–	Six were shown to be (2S)-2-hydroxycurdione, (2R)-2-hydroxycurdione, (8S)-6-hydroxycurdione, (2R, 8S)-8-hydro-2-hydroxycurdione, (1S, 10S)-1,10-epoxycurdione and (1R, 10R)-1,10-epoxycurdione in suspension cultured cells induced from calli of *L. japonica.*	Horiike and Ohshiro (1997)
1999	Stem	MS	10 µM NAA+10 µM BA	These cultures were agitated on a rotary shaker at a speed of 100 rpm min^-1^ at 23°C in the dark and subcultured every 2-3 weeks for over 1 year.	The cells also converted 7-deoxyloganin into both loganin and secologanin.	Yamamoto et al. (1999)
2003	Zygotic embryo	MS	4.52 mM 2,4-D	The flask was placed on a gyratory shaker at 100 rpm, all cultures were incubated at 25°C in the dark.	A cell suspension culture system was established.	Kim et al. (2003)
2010	Flower bud	MS	1.5 mg/L BA+0.75 mg/L KT+0.5 mg/L NAA	The temperature was 25 ± 2°C, with a light duration of 14 h/d and a light intensity of 2000 lx. The culture was placed in a shaking incubator at 120 r·min^−^¹ for suspension culture.	During the culture process, the dry weight of the cells showed an increasing trend, reaching a maximum of 8 g/L at 18 d. The soluble sugar content increased rapidly, peaking at 18 d. Soluble protein, POD, and SOD activities peaked at 12 d.	Liu (2010)
2012	Young leaf	MS	2.0 mg/L 2,4-D+0.5 mg/L KT	The culture was placed in a shaker incubator at 100 r·min^−^¹ and 26°C under dark conditions.	Under liquid suspension culture conditions, the cell growth cycle lasted 14 d.	Wu (2012)
2016	Bud	MS	1.4 mg/L 6-BA+0.5 mg/L NAA+0.7 mg/L 2,4-D	The culture was subjected to shaking at 110 r·min^−^¹ and 28°C under dark conditions for 7 d.	The growth of honeysuckle suspension cells peaked on day 10. High-performance liquid chromatography (HPLC) analysis revealed that each 100 g of dry cells contained 826.7 mg of CGA.	Hu (2016)
2016	Young leaf	B5	2.0 mg/L 6-BA+2.0 mg/L 2, 4-D	The cultures were kept under continuous agitation at 110 r·min^−^¹ in an orbital shaker and incubated at 25°C, with a 16 h photoperiod (40 μmol m^-2^ s^-1^).	The findings provide a potential basis for large scale production of CGA using cell suspension cultures of *L. macranthoides.*	Li et al. (2016)
2019	10- day-old buds; 15-day-old young leaves; 15-day-old young stem	MS	1.5 mg/L 6-BA+0.2 mg/L NAA+0.1 mg/L 2, 4-D	The inoculated flasks were placed on a rotary orbital shaker at 110 r·min^−^¹ and incubated at 28 ± 2°C in the dark.	A cell suspension system was successfully established, with characteristic of a “S” growth curve and healthy morphology. 3,5-di-O-caffeoylquinic acid, 3-O-caffeoylquinic acid, 4,5-di-O-caffeoylquinic acid and 3,4-di-O-caffeoylquinic acid were the main secondary metabolites in the suspension culture cells. The total content of these CGAs was 22.7 mg g^-1^ in the suspension cells.	Hu et al. (2019)
2019	Young leaf	MS	0.6 mg/L 6-BA+0.5 mg/L NAA+0.2 mg/L 2,4-D	The culture was shaken at 110 r·min^−^¹ and 26°C in a shaker incubator.	The culture was shaken at 110 r·min^−^¹ and 26°C in a shaker incubator.	Zu et al. (2019)
2022	Stem	MS	0.2 mg/L 6-BA+0.8 mg/L NAA	All cells were subsequently cultured in a shaker at 110 r·min^−^¹, and at a temperature of 22 ± 2°C.	The cell growth curves were determined using the optimal cell suspension system described above. Cell growth peaked at 18 d of suspension culture, with the fresh weight of cells being 676.0 ± 12.66 g/L.	Wang et al. (2022)

### 2.4 Culture of *L. japonica* protoplasts

Plant protoplasts refer to naked cells that retain cellular totipotency after the removal of the cell wall (Avila-Peltroche et al., 2020). As an important tool in modern plant biology, protoplasts serve not only as ideal recipient materials for foreign gene introduction but also as an effective means for somatic hybridization to develop new cultivars (Seidel et al., 2024). Liu et al. (2012a) used vigorously growing and loosely structured *Lonicera* callus as material for protoplast isolation. The enzymatic solution was prepared with 1.5% cellulase and 0.25% pectinase, supplemented with 0.7 mol/L mannitol as an osmotic stabilizer in CPW (cell-protoplast washing solution). The enzymatic digestion was performed under static conditions at 25°C for 12 h, yielding a large quantity of high-quality protoplasts.

### 2.5 Research progress of the hairy roots culture of *L. japonica*

The transformation of hairy roots and their tissue and organ culture offers multiple advantages, including genetic and biochemical stability, rapid growth, independence from exogenous plant hormones, and the ability to stably produce active compounds. Furthermore, as hairy roots originate from single cells, they facilitate extensive clonal selection, making the use of hairy root cultures for high-yield secondary metabolite production a prominent research focus worldwide (Sharma et al., 2013; Kundu et al., 2018). The results showed that when the Agrobacterium rhizogenes R1000 culture reached an OD600 value of approximately 0.6 and was diluted 40-fold before immersing explants for 4 minutes, the induction rate was highest, reaching 58%. Pre-culturing explants for 5 days, followed by co-cultivation with Agrobacterium for 3 days and subsequent transfer to a medium containing 500 mg/L carbenicillin for sterilization, was identified as the key condition for achieving optimal induction (Yuan and He, 2007). He and Zhou (2008) further optimized the induction conditions for *L. japonica* hairy roots. Their results indicated that leaf explants from tissue culture seedlings, after 5 days of pre-cultivation, immersion in bacterial suspension diluted 45-fold for 4 minutes, and 3 days of co-cultivation, followed by transfer to 1/2 MS medium containing 500 mg/L carbenicillin, achieved the highest induction rate of 54.3%. The successful establishment of *L. japonica* hairy roots provides an important foundation and evidence for the construction of Ri plasmid-mediated genetic transformation systems and the industrial-scale production of medicinal active compounds through hairy root cultures.

### 2.6 Polyploid induction of *L. japonica*

Compared to diploid plants, polyploid plants generally exhibit greater vigor, yield, and quality, as well as enhanced resistance to various biotic and abiotic stresses. Additionally, polyploid breeding can overcome the incompatibility issues of distant hybridization, making it an important approach for germplasm innovation in medicinal plants (Schatlowski and Köhler, 2012; van Berkum et al., 2010). Liu et al. (2012b) induced tetraploid plants from *L. japonica* “Hongxing No. 2” callus tissues by soaking them in 0.1% colchicine for 15 h. Compared to diploid plants, the tetraploid plants exhibited deeper leaf color, slower growth, shorter and thicker internodes, as well as significantly enlarged stomata and guard cells with reduced stomatal density. Wang and Zhao (2012; 2014) used a modified L.D. Cua method to artificially mutate diploid *L. japonica*. Cytological and early morphological analyses of the M1 mutant plants revealed that while the control plants were diploid (2n = 2x = 18), the M1 mutant plants were tetraploid (2n = 4x = 36). The tetraploid plants exhibited enhanced growth vigor, deeper leaf color, larger and thicker leaves, and a reduced leaf shape index after chromosome doubling. “Jiufeng No. 1”, a homologous tetraploid bred using polyploid breeding techniques, was developed from the traditional *L. japonica* cultivar “Damaohua” from Pingyi. Compared to its parent “Damaohua”, its CGA content increased by 30%, and its average yield improved by 58.79% (Xing, 2006). Zhang et al. (2011) induced allopolyploid lines of hybrid *L. japonica* under *in vitro* tissue culture conditions. Field agronomic observations showed that the flower bud size of the polyploid lines was significantly larger, and the average CGA content in the allopolyploid lines was 1.34 times that of the hybrid diploid control (CK).

## 3 Research on the metabolic regulation of CGA in *L. japonica*

The synthesis of CGA originates from the complex phenylpropanoid metabolic pathway (Hu et al., 2024; Pan et al., 2021b). Inducers are substances capable of activating or enhancing the synthesis of secondary metabolites in plants (Petrova et al., 2024; Bhaskar et al., 2022). Under the influence of inducers, plants undergo stress responses characterized by the activation of defense-related genes or the inactivation of non-defense-related genes, transient protein phosphorylation or dephosphorylation, and the expression of related enzymes. These molecular changes provide critical insights into the biosynthetic pathways of secondary metabolites (Narayani and Srivastava, 2017; Yamaner et al., 2013).

### 3.1 Metabolic pathway of CGA biosynthesis

**Figure 4:**
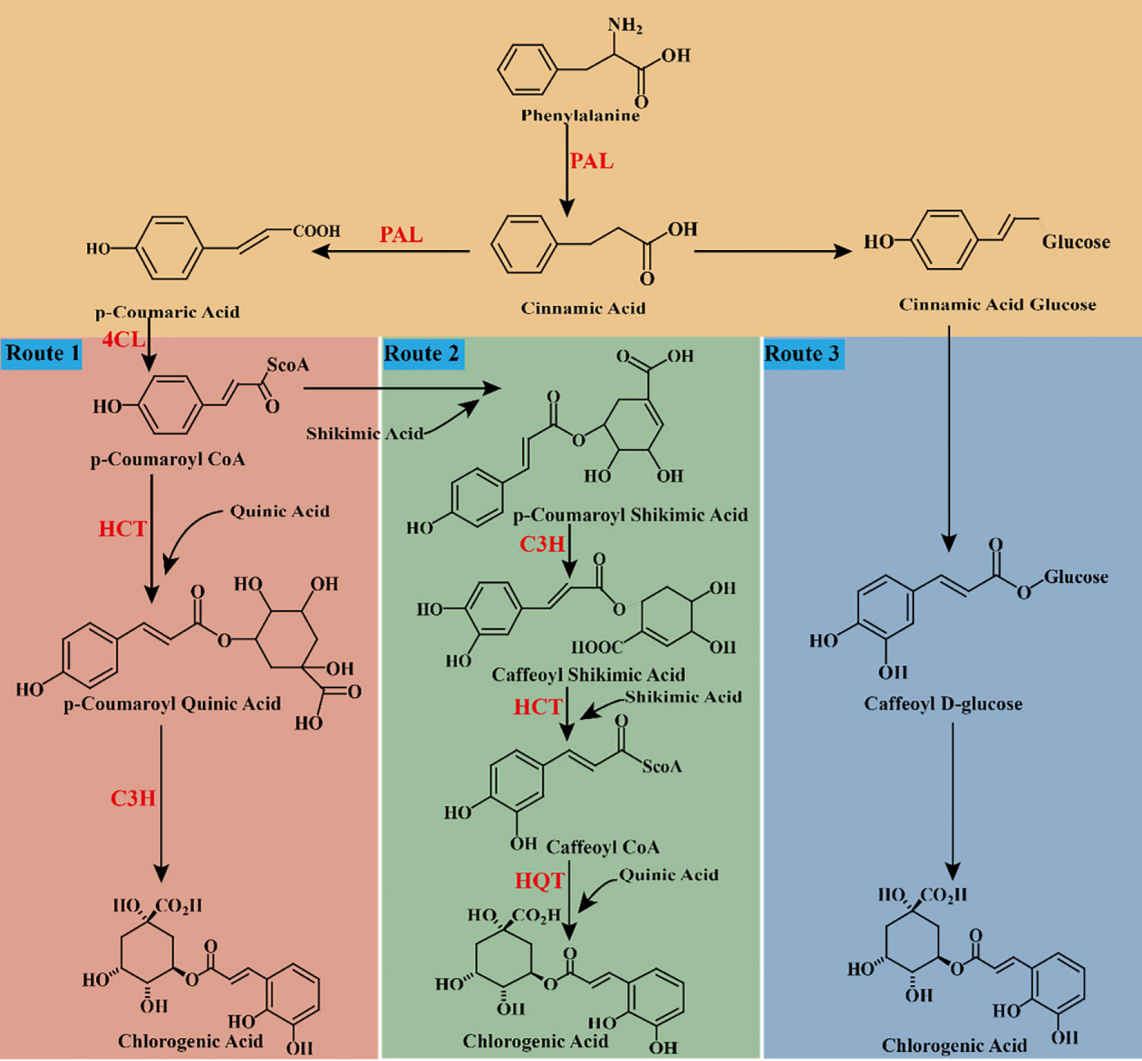
chematic cartoon displays 3 synthetic pathways of CGA. PAL: phenylalanine ammonia-lyase. C4H: cinnamate-4-hydroxylase. 4CL: 4-coumarate-CoA ligase. C3H, coumaric acid-3-hydroxylase; HCT, shikimate/quinate hydroxycinnamoyl transferase; HQT, quinate hydroxycinnamoyl transferase.

The synthesis of CGA originates from the complex phenylpropanoid pathway. Based on existing literature, three main biosynthetic pathways for CGA have been summarized (**Figure 4**). Initially, glucose in plants is catalyzed by specific enzymes to form shikimic acid, which is further converted into phenylalanine. Subsequently, through the sequential catalysis of phenylalanine ammonia-lyase (PAL), cinnamate-4-hydroxylase (C4H), and 4-coumarate-CoA ligase (4CL), phenylalanine is transformed into p-coumaroyl-CoA. p-Coumaroyl-CoA acts as a precursor for the biosynthesis of flavonoids and lignin, and it also represents a critical starting point for the CGA biosynthetic pathway. (1) Route 1: p-Coumaroyl-CoA reacts with quinic acid (QA) under the catalysis of hydroxycinnamoyl-CoA shikimate/quinate hydroxycinnamoyl transferase (HCT) to produce p-coumaroyl quinic acid, which is subsequently hydroxylated by coumarate-3-hydroxylase (C3H) to form CGA (Valiñas et al., 2015) (**Figure 4**, Route 1). (2) Route 2: p-Coumaroyl-CoA combines with shikimic acid to form p-coumaroyl shikimic acid, which is hydroxylated by coumaric acid-3-hydroxylase (C3H) to produce caffeoyl shikimic acid. Subsequently, caffeoyl-CoA is synthesized by hydroxycinnamoyl-CoA shikimate/quinate hydroxycinnamoyl transferase (HCT). Finally, caffeoyl-CoA reacts with quinic acid under the catalysis of hydroxycinnamoyl-CoA: quinate hydroxycinnamoyl transferase (HQT) to form CGA via esterification (Tang et al., 2021; Niggeweg et al., 2004) (**Figure 4**, Route 2). (3) Route 3: Caffeoyl glucose is converted into CGA by quinic acid hydroxycinnamoyl transferase (Tang et al., 2021; Niggeweg et al., 2004) (**Figure 4**, Route 3).

### 3.2 The function of key rate-limiting enzyme genes in the CGA synthesis pathway

Studies have shown that HQT (Luo et al., 2018; Zhang et al., 2016; Wu et al., 2014; Liu et al., 2012c), PAL (Wu et al., 2024; Li et al., 2024a), 4CL (Li et al., 2015), HCT (Chen et al., 2024; He et al., 2013), and C3H (Chen et al., 2020) are key enzymes in the biosynthetic pathway of CGA. These enzymes play a crucial role in the biosynthesis of CGA in *L. japonica*.

PAL is a key bridge between primary and secondary metabolism, catalyzing the conversion of phenylalanine to cinnamic acid. It controls the metabolic flux of precursors into the phenolic pathway and plays an essential role in regulating the overall level of accumulated phenolic compounds (Pan et al., 2021b; Clé et al., 2008) Therefore, PAL plays a crucial role in plant growth and development, as well as in responding to biotic and abiotic stress defenses, such as mechanical damage and ultraviolet radiation (Zhao et al., 2022). C4H is the second key rate-limiting enzyme in the phenylpropanoid pathway, localized to the endoplasmic reticulum of plant cells. It belongs to the cytochrome P450 enzyme family, specifically the CYP73 subgroup (Zhang et al., 2020). As a P450 protein, C4H catalyzes an irreversible reaction, using NADPH as an electron donor in the presence of oxygen and reduced nicotinamide adenine dinucleotide phosphate (NADPH). This enzyme catalyzes the conversion of trans-cinnamic acid to p-coumaric acid (Cheng et al., 2018). 4CL is a major branch point for cinnamic acid derivatives, converting them into different types of coenzyme A derivatives, including p-coumaric acid, caffeic acid, ferulic acid, and cinnamic acid. These intermediates are the precursors for synthesizing phenylpropanoid compounds such as lignin, CGA, and flavonoids (Li et al., 2015; Li and Nair, 2015). C3H is another key enzyme in the CGA biosynthesis pathway and the second hydroxylase in the biosynthesis of fatty acids, caffeic acid, and other phenolic acids. It belongs to the CYP98A family of cytochrome P450 monooxygenases (Yang et al., 2020). HCT belongs to the BAHD acyltransferase family and contains two conserved structural domains, HXXXD and DFGWG, which are present in all terrestrial plants (Yang et al., 2003). In the phenylpropanoid metabolic pathway, HCT is located at both the upstream and downstream junctions of the hydroxylation process catalyzed by C3H, enabling it to regulate the target genes of both the upstream and downstream pathways. It not only generates p-coumaroyl shikimate/quinic acid as substrates for C3H but also converts the products generated by C3H, caffeoyl shikimate/quinic acid, into caffeoyl-CoA. Therefore, HCT is considered to play a dual role in the phenylpropanoid hydroxylation pathway (Chao et al., 2021). HQT also belongs to the BAHD acyltransferase family and contains the two gene sequences HXXXD and DFGWG (Yang et al., 2003). HQT is tightly linked with HCT. Unlike the substrate versatility of HCT, HQT has substrate specificity and is the key rate-limiting enzyme in the final step of CGA biosynthesis. It catalyzes the ester exchange between caffeoyl-CoA and quinic acid, producing structurally different CGA derivatives (Li et al., 2019).

### 3.3 Elicitors

**Figure 5:**
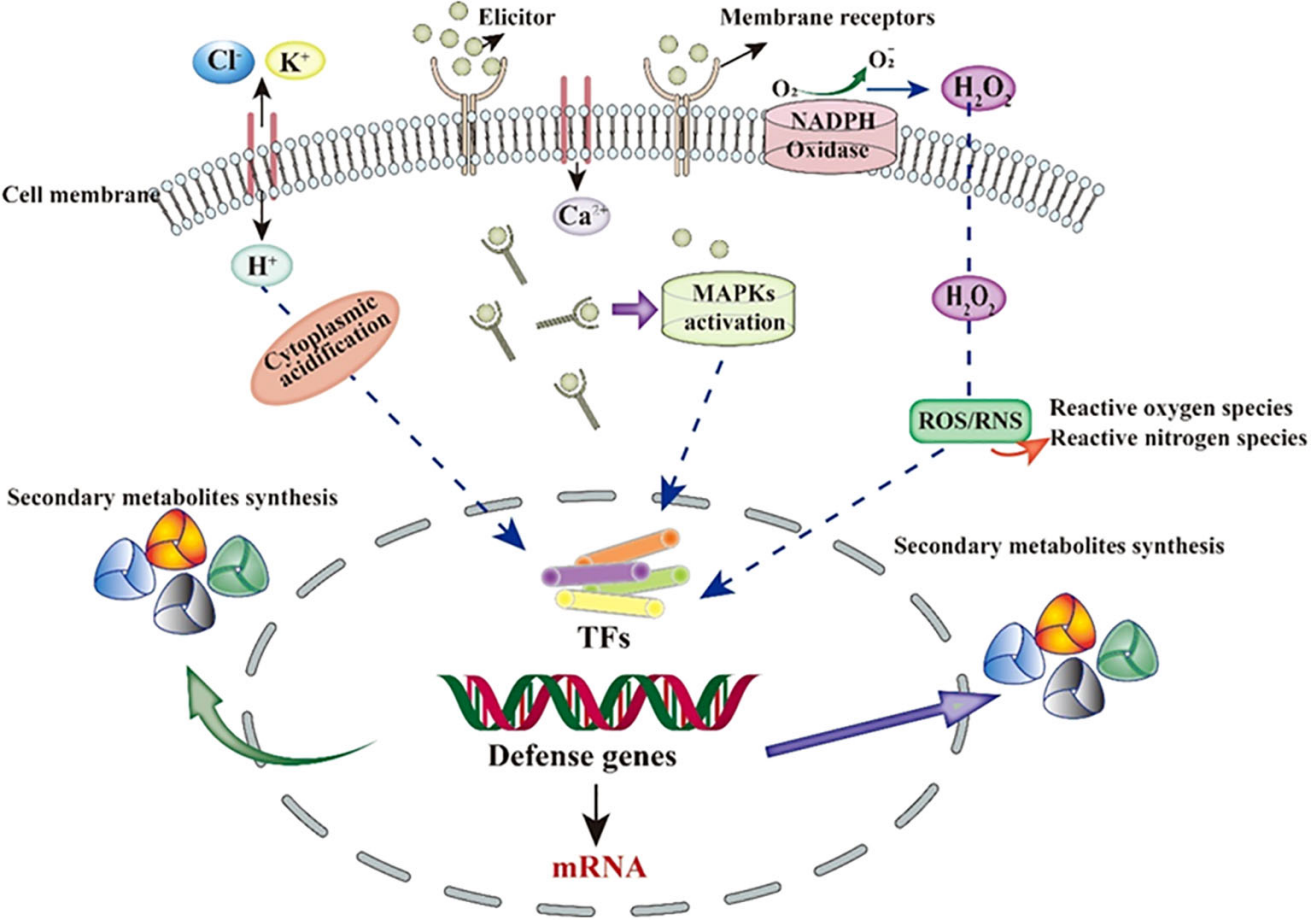
Schematic representation of the general mechanism of biotic elicitors.

Inducers are a class of chemical substances or biological factors that promote the production of target metabolites and induce physiological changes in plants (Petrova et al., 2024; Hatami et al., 2019). Studies have shown that adding inducers can effectively reduce cultivation costs, increase cultivation efficiency, promote the production of medicinal plants, and enhance the yield of secondary metabolites, ultimately boosting the production of medicinal plants (Bhaskar et al., 2022; Narayani and Srivastava, 2017; Thakur et al., 2020). Inducers come in a wide variety of types and can be classified based on their origin into biological inducers, abiotic inducers, and novel nanoparticle inducers (Hussain et al., 2022). The transduction of induction signals within plants is mediated by multiple parallel or interconnected signaling pathways. These pathways establish an effective defense system in response to various external stimuli, leading to different reactions both inside and outside the plant (Zhang et al., 2012; Malik et al., 2011). Generally, the induction effect of inducers in plants can be divided into four steps: first, the induction signal is recognized and bound by receptors on the cell membrane; next, ion channels across the cell membrane are altered; then, the second messenger transduces the signal to the cell nucleus, activating gene regulatory factors; and finally, specific biological effects are generated in the plant (Baenas et al., 2014). The interaction between the inducer molecules and specific receptors on the surface of plant cell membranes is the most critical step in the action of inducers (Ferrari, 2010; Shasmita et al., 2018). After the induction signal is sensed, ion channels on the cell membrane are altered, leading to an increase in cytoplasmic Ca^2+^, efflux of Cl^-^/K^+^, and influx of H^+^, resulting in cytoplasmic acidification. Subsequently, the second messenger transduces the inducer signal into the cell (Abdul Malik et al., 2020; Ludwig et al., 2004; Shabala and Pottosin, 2014). Inside the cell, mitogen-activated protein kinases (MAPKs) and reduced coenzyme II (NADPH) are activated, producing reactive oxygen species (ROS) and reactive nitrogen species (RNS) (Zhao et al., 2005; Mishra et al., 2012). Ultimately, regulatory factors within the cell are activated, regulating the expression of intracellular defense genes. This may directly or indirectly lead to the synthesis of secondary metabolites to resist changes in the external environment (as shown in **Figure 5**).

### 3.4 Effect of inducers on CGA of *L. japonica*

**Figure 6:**
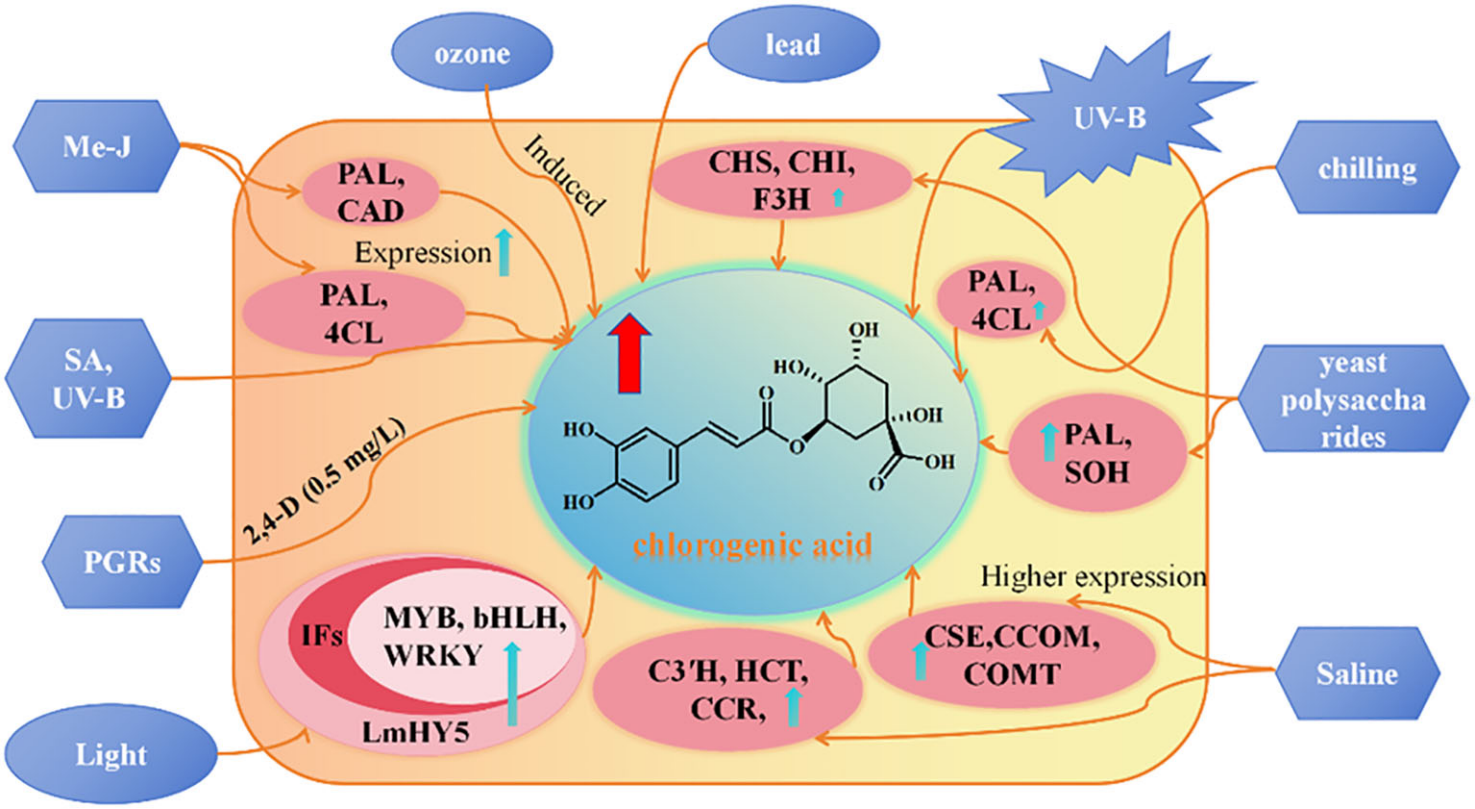
Study on the regulation of secondary metabolites of *L. japonica*. Note: The blue boxesrepresent biological and abiotic elicitors, the pink boxes represent genes, and the brown boxesrepresent secondary metabolite types. methyl jas-monate (MJ), salicylic acid (SA), chalcone synthase (CHS), chal-cone isomerase (CHI), phenylalanine ammonia (PAL), cinnamic acid 4-hydroxylase (C4H), Cinnamyl alcohol dehydrogenase (CAD), 4-coumarate-CoA ligase (4CL), flavanone 3-hydroxylase (F3H), caffeoylshikimate esterase (CSE), caffeoyl-CoAO-methyltransferase (CCOM), caffeic acid 3-O-methyltransferase (COMT), candidate catalytic enzymes (C3′H), hydroxycinnamoyl transferase (HCT), cinnamoyl-CoA reductase (CCR), Light sig-nalling of *L. macranthoides* (LmHY5), transcription factors (TFs).

To enhance the CGA content in *L. japonica* as in **Figure 6**, Gu et al. (2024) proposed an induction strategy based on yeast polysaccharide and revealed its induction mechanism. The study revealed significant differences in active component content between the 0.1 g/L yeast polysaccharide treatment group and the control group. Transcriptome analysis identified 218 differentially expressed genes, with 60 upregulated and 158 downregulated. Further analysis revealed 12 key genes involved in the biosynthesis of phenolic compounds, including PAL1, PAL2, PAL3, 4CL1, 4CL, CHS1, CHS2, CHS, CHI1, CHI2, F3H, and SOH, of which SOH being a specific gene for the synthesis of caffeic acid and CGA. Methyl jasmonate (MeJA), salicylic acid (SA), and ultraviolet radiation (UV) can significantly induced and regulated the synthesis of CGA compounds in *L. japonica* suspension-cultured cells. Under conditions of MeJA at 200 μmol/L, SA at 50 μmol/L, UV exposure for 2 h, and a 5-day induction period, the total CGA content in suspension cells reached 4.6 times that of the control group. Further transcriptome analysis revealed that, after MeJA induction (200 μmol/L) for 4, 10, and 20 h, the expression levels of key CGA biosynthetic enzyme genes: C4H, PAL, HCT, C3′H, CAD, and 4CLwere significantly upregulated compared to the control (0 h) (Li, 2018). Ecological conditions play a critical role in the accumulation and synthesis of secondary metabolites in medicinal plants, including altitude, temperature, light intensity, and light quality (Li et al., 2020). Chen et al. (2023) found that, after 7 days of shading treatment, CGA content in *L. japonica* leaves significantly decreased by 1.78-fold. Concurrently, the overall transcriptional expression of CGA biosynthetic pathway-related genes was downregulated, while the expression of photosynthetic signal protein-related genes was upregulated. During the light signaling transduction process, the transcriptional expression of PHOT, HY5, and PIF was significantly influenced by shading treatment, regulating the expression of transcription factors (TFs) such as MYB, bHLH, and WRKY. These regulatory effects collectively led to a reduction in CGA content. Moreover, it was demonstrated that the transcriptional expression of HY5 in *L. japonica* leaves positively correlates with CGA content, confirming that decreased light intensity downregulates HY5 expression, which is a key factor contributing to the reduction in CGA levels. Salt-alkali stress can also promote the accumulation of phenolic compounds in *L. japonica* leaves. Yan et al. (2017) demonstrated that NaCl stress activates the synthesis of phenolic compounds, as evidenced by a significant increase in the transcription level and activity of the PAL gene, accompanied by an elevated concentration of phenolic compounds. After 15 days of 150 and 300 mM NaCl stress, the CGA content in leaves increased by 67.43% and 48.86%, respectively, while the luteoloside content increased by 54.26% and 39.74%, respectively. Cai et al. (2021) further revealed that the synthesis of phenolic acid metabolites in *L. japonica* under NaCl stress is closely related to the expression of upstream enzymes in the phenylalanine metabolism pathway, including PAL, C4H, and 4CL. Additionally, HCT, C3′H, and COMT were significantly upregulated during phenolic acid synthesis, while FLS and CHI showed marked increases in expression during flavonoid biosynthesis. Zhang et al. (2022a) found that after 30 days of 10°C low-temperature stress, the CGA and luteoloside contents in *L. japonica* significantly increased, reaching 23 times and 17 times that of the control group, respectively. Simultaneously, the expression of CGA biosynthesis-related genes PAL and 4CL was significantly upregulated under low-temperature stress, while the upregulation of CHS and CYP75B1 activated the luteoloside biosynthesis pathway. These findings suggest that low-temperature stress promotes the accumulation of metabolites by regulating the phenylalanine metabolism and associated secondary metabolic pathways. The inducers on CGA content in *L. japonica* are summarised in **Table 4**.

**Table 4:** Effect of Inducer on CGA of *L. japonica*.

Material	Activity Component	Influence Factor	Metabolic Regulation	Result
Leaf	CGA concentration; Luteolosid concentration	Saline stress (150 mM, 300 mM NaCl, 15 d)	PAL gene family consists of PAL1, PAL2 and PAL3, and their transcription was significantly elevated by 150 and 300 mM NaCl at day 7 and 15 d.	Genes transcription and activity of phenylalanine ammonia-lyase and increased phenolics concentration. After 15 d of NaCl stress at 150 mM and 300 mM, the CGA content in leaves increased by 67.43% and 48.86%, respectively, while the luteoloside content increased by 54.26% and 39.74% (Yan et al., 2017).
Leaf	CGA concentration; Luteolosid concentration	Chilling stress (10°C, 30 d)	Cold stress activated secondary metabolic pathways such as phenylpropanoid-flavonoids and carotenoids. The PAL and 4CL genes in the phenylalanine metabolism pathway, involved in CGA biosynthesis, were upregulated under cold stress, promoting the synthesis of CGA. In the carotenoid biosynthesis pathway, genes such as DXR, ISPF, GGPS, GGPPS, PDS, ZDS, LCYE, and LCYB were significantly activated, driving the synthesis of carotenoids.	The contents of CGA and verbascoside both showed an increasing trend under cold stress. From the starting point (SP) to 30 d of treatment (T30), the contents of both compounds gradually accumulated, with a significant increase at T30. Compared to SP, the CGA content increased 23-fold, and the verbascoside content increased 17-fold (Zhang et al., 2022a).
Flower	Phenylpropanoid, flavonoid, and iridoid	Salt stress	PAL, C4H, and 4CL were significantly upregulated, promoting the synthesis of phenolic acid-derived secondary metabolites. CHI is involved in flavonoid synthesis and its expression was upregulated under salt stress, affecting the synthesis of flavonoid secondary metabolites. The expression of the F3H gene varied in the salt treatment group, suggesting that the changes in flavonoid levels may be jointly regulated by upstream and downstream pathways. This influenced the synthesis and accumulation of flavonoid secondary metabolites. DXS and CYP72A1 were upregulated under salt stress, driving the synthesis and accumulation of sesquiterpenoid compounds.	15 bioactive constituents (7 phenolic acids, 4 flavonoids, and 4 iridoids), which were significantly accumulated (Cai et al., 2021).
Leaf	The content of organic acids, flavonoids and iridoids	Yeast polysaccharides (0.1 g/L, 1 g/L, 5 g/L and 10 g/L; 6 d)	The expression of PAL1, PAL2, and PAL3 was significantly increased, regulating the phenylpropanoid biosynthetic pathway and influencing the synthesis of downstream secondary metabolites. The expression of 4CL1 and 4CL showed dynamic changes, playing an important regulatory role in the phenylpropanoid biosynthetic pathway. The expression of CHS1, CHS2, and CHS was significantly upregulated, having a crucial impact on flavonoid compound synthesis. The expression of the SOH gene promoted the synthesis of caffeic acid and CGA.	Low concentrations of yeast polysaccharides had a significant impact on organic acid components. The contents of caffeic acid, CGA, and chlorogenic acid A significantly increased the day after application. The growth effect of coumaric acid was optimal 5 days after treatment with 0.1 g/L yeast polysaccharides. Verbascoside showed the most significant increase 5 days after treatment with 0.1 g/L yeast polysaccharides. Low concentrations of yeast polysaccharides significantly enhanced the levels of secoxyloganin, sweroside, and morroniside (Gu et al., 2024).
Suspension cell	CGAs content	200 μM Me-J, 50 μM SA and 2 h/d UV-B	—	The optimal inducer combination significantly promoted the accumulation of four types of CGAs. On the sixth day of treatment, the total CGA content reached its maximum, increasing 4.52-fold and 3.13-fold compared to the control group and field flower buds, respectively (Du et al., 2020).
Stem, leaf & flowe*r*	Polysaccharide, total polyphenols, total flavonoid, and CGA	UV-B (8.4, 22.4 μW/cm²; 2, 4, 6, 8 h)	—	The contents of polysaccharide, total polyphenols, total flavonoid, and CGA in different organs all increased significantly and reached a peak under the UV-B (8.4 μW/cm²) treatment for 2 h (Wu et al., 2021).
Suspension cell	Production of CGA	PGRs	—	When the concentration of 6-BA was 2.0 mg/L, both biomass and CGA accumulation were higher. In the hormone combination, 2.0 mg/L 6-BA+0.5 mg/L 2,4-D was beneficial for CGA accumulation, and 2.0 mg/L 6-BA+2.0 mg/L 2,4-D favored biomass accumulation (Li et al., 2016).
Leaf	The content of flavonoids and CGA	Cadmium addition in soil and ozone stress	In the comparison between CK and Cd, the MAPK signaling pathway-plant pathway showed the highest enrichment significance in honeysuckle leaves. In the comparison between CK and EO, the photosynthesis pathway showed the highest enrichment significance; In the comparison between EO and EO-Cd, the photosynthetic antenna protein pathway exhibited the highest enrichment significance.	Cadmium addition in soil and ozone stress significantly inhibited the CGA content but the content of flavonoids was increased (Tao, 2023).
Suspension cell	The total content of CGAs	200 µM Me-J	C4H, PAL, HCT, C3’H, CAD, 4CL were up-regulated with higher differential expression folds.	The total content of CGAs reached up to 6.99% (Li, 2018).
Leaf	The content of free proline, soluble sugar and soluble protein	Lead induction (0, 400, 600, 800, 1000 mg/kg)	—	Under lead stress, the chlorophyll content in honeysuckle decreased as the lead concentration increased, while the levels of free proline, soluble sugars, and soluble proteins increased, with the most significant changes observed at a lead concentration of 2800 mg/kg. Lead stress also led to an increase in malondialdehyde (MDA) and antioxidant enzyme activities. At a lead concentration of 600 mg/kg, MDA content showed no significant difference from the control group, but SOD activity increased by 41.65%. When the lead concentration reached 1000 mg/kg, MDA content peaked, with a significant difference compared to the control group, and POD and CA activities increased by 78.92% and 45.60%, respectively (Mao et al., 2019).
Flower	Isochlorogenic acids and iridoid glycoside	Ultraviolet induction	—	The contents of isochlorogenic acid in *L. japonica* increased significantly. Secologanic acid, secoxyloganin, secologanin and (E)-aldosecologanin are all iridoid compounds, and their contents all increased significantly after ultraviolet radiation (Ning, 2012).

## 4 Conclusion and prospects

### 4.1 Conclusion

Tissue culture technology, as an essential component of modern plant biotechnology, holds significant application potential. It not only provides technological support for the efficient utilization of medicinal plant resources and the production of active ingredients, but also offers innovative solutions for plant breeding, genetic improvement, and resource conservation. With the continuous development of biotechnology, the integration of tissue culture technology with other modern biotechnologies is expected to drive the efficient and sustainable development of the medicinal plant industry, providing richer resources and technological support for global sectors such as pharmaceuticals and agriculture. This review summarizes the latest research progress on the *in vitro* culture techniques of *L. japonica* and explores the application and impact of elicitors in the CGA biosynthesis pathway. *In vitro* culture techniques, such as direct organogenesis, callus culture, suspension cell culture, protoplast culture, hairy root culture, and polyploid induction, provide various potential pathways for the production of secondary metabolites and resource conservation in *L. japonica*. Regarding the biosynthesis of CGA, this review also discusses the key enzyme genes involved in the process, including PAL, C4H, 4CL, C3’H, HCT, and HQT, which play critical roles in the metabolic pathway of *L. japonica.*

### 4.2 Prospects

Although current research has revealed the potential of *L. japonica in vitro* culture techniques for CGA synthesis, future studies still face several challenges and research directions.

Firstly, breakthroughs in anther culture techniques. During the literature review, no previous research on the anther culture of *L. japonica* via the indirect organogenesis pathway was found. Compared to other explant culture methods, anther culture can directly form haploid plants from pollen, which can then be converted into homozygous plants through polyploid induction. The resulting homozygous plants exhibit higher genetic stability due to identical alleles at each locus in their genome. It can also accelerate germplasm innovation by selecting and cultivating specific pollen to obtain plants with particular traits or superior genes. More importantly, anther culture provides a powerful platform for genomics research and genetic improvement. By combining gene editing technologies, such as CRISPR/Cas9, precise gene manipulation can be conducted during the anther culture process, further advancing molecular breeding and genomic selection applications in plants. Furthermore, when combined with molecular marker techniques, anther culture can more accurately select plants with desired traits, thus improving breeding efficiency. Therefore, it is evident that the anther culture technology for *L. japonica* needs to be advanced.

Secondly, in-depth study of the regulatory mechanisms of elicitors. Although elicitors have been confirmed to promote the synthesis of CGA, the molecular mechanisms by which they regulate the expression of key enzyme genes through signal transduction pathways remain poorly understood. Future research should focus on how elicitors regulate the expression of key enzyme genes in the CGA biosynthesis pathway through pathways such as MAPK signaling, ROS production, and Ca^2+^ signaling, to reveal their complex regulatory mechanisms.

Thirdly, challenges in the Scalable Production of *In Vitro* Culture Systems. Tissue culture technology is one of the application areas of plant biotechnology that allows the extraction of valuable plant metabolites under controlled conditions. Compared to traditional methods, the production of secondary metabolites through plant cell and tissue culture offers significant advantages. These advantages include the ability to produce target metabolites under controlled conditions, independent of environmental factors, and the potential to optimize culture conditions to enhance secondary metabolite yields. the ability to provide adequate yields, if necessary, taking into account the balance between supply and demand, and thus to regulate the market on a regular basis. Additionally, *in vitro* culture can help balance supply and demand by ensuring sufficient production when needed, allowing for market regulation. It also offers a rapid production rate that is unaffected by political constraints. Furthermore, it enables the production of disease-free and contaminant-free plant materials, and any plant species, regardless of whether they originate from tropical or subtropical regions, can be cultivated *in vitro*. Although *in vitro* culture technology has a high potential for metabolite accumulation, it still faces several technical challenges in large-scale production, such as standardization of culture conditions, medium cost, contamination control and production efficiency. In order to overcome these challenges and maximize its potential, future research needs to focus on optimizing scale-up strategies for *in vitro* culture systems, improving the balance between cell growth and metabolism, reducing production costs while enhancing economic viability, and exploring the application of gene editing and regulatory approaches in large-scale production.

Fourthly, comprehensive optimization of *in vitro* culture technology. Different *in vitro* culture techniques, such as direct organogenesis, callus culture, suspension cell culture, protoplast culture, hairy root culture, and polyploid breeding, each have their own advantages. However, there is a lack of detailed mechanistic studies on how these techniques influence the biosynthetic pathway of CGA at the molecular level. Future research could integrate advanced technologies such as transcriptomics, proteomics, and metabolomics to comprehensively analyze the regulatory mechanisms by which different culture techniques influence CGA biosynthesis. This would help elucidate the intricate regulatory effects of various culture methods on secondary metabolite synthesis in plants, providing a theoretical foundation for optimizing culture conditions at an industrial scale to enhance CGA yield. Additionally, studies should explore how culture techniques affect CGA biosynthesis by modulating plant signaling pathways, hormone levels, and stress responses. Building on this foundation, the integration of molecular biology and gene editing technologies may enable precise regulation of plant secondary metabolism, thereby improving the efficiency and stability of CGA production.

Fifthly, exploration of Genotypic Differences. Current research primarily focuses on a single genotype of *L. japonica*, whereas different genotypes may respond differently to *in vitro* culture conditions and elicitors. Different genotypes may exhibit significant variations in response to *in vitro* culture conditions and elicitors, and these differences may lead to distinct accumulations of bioactive compounds by regulating the expression of key enzyme genes in secondary metabolic pathways. Comparative studies across multiple genotypes can help identify superior germplasm with high yields of specific metabolites, providing a stable raw material supply for pharmaceutical development. Significant differences in the response of different genotypes of *L. japonica* in tissue culture underscore the importance of including multiple genotypes in research. This approach helps comprehensively evaluate the universality and specificity of key culture parameters (such as explant selection, sterilization methods, and hormone ratios) across different genotypes, providing theoretical foundations and technical support for establishing more efficient and standardized culture systems. Research on multi-genotype tissue culture can identify genotypes with faster growth and stronger metabolic product synthesis capabilities, and by combining rapid propagation techniques, it can achieve sustainable resource utilization. Furthermore, based on research data from different genotypes, a “genotype-phenotype-metabolome” database for *L. japonica* can be established, supporting precision breeding and the development of functional products. Therefore, it is crucial to include multiple genotypes of *L. japonica* in future research to conduct in-depth studies on tissue culture and secondary metabolite biosynthesis pathways.
